# ICU outcome of patients undergoing cytoreductive surgery followed by hyperthermic intraperitoneal chemotherapy: a single-center study

**DOI:** 10.1186/cc14456

**Published:** 2015-03-16

**Authors:** A Nadeem, A Al-Tarifi

**Affiliations:** 1 King Faisal Specialist Hospital, Riyadh, Saudi Arabia

## Introduction

Peritoneal carcinomatosis (PC) is associated with poor prognosis. The advent of complete cytoreductive surgery (CRRS) followed by hyperthermic intraperitoneal chemotherapy (HIPEC) has shown promise in improved survival for locally advanced intraabdominal carcinomatosis. Such patients are routinely admitted to the ICU postoperatively. Little is known about the natural course of such patients while in the ICU.

## Methods

The procedure was introduced in our hospital in 2008 as the first regional center performing such therapy. A retrospective chart review of 129 cases of CRS-HIPEC admitted to a 22-bed surgical ICU in a tertiary care academic center between November 2008 and March 2014. Primary outcomes were ICU length of stay (LOS) and duration of mechanical ventilation (MV). Secondary outcomes were hospital LOS and hospital mortality.

## Results

Eighty-seven patients (69.9%) were females. Mean age was 48.9 years. Primary cancer was colorectal in 42 patients (32.5%), ovarian in 39 (30%), appendiceal in 29 (22%), others in 15.5%. Average operative time was 11 ± 2.1 hours. Average intraoperative crystalloids given were 12,217 ± 4,359 ml, packed RBCs were 2 ± 2.3 units, colloids 1,083 ± 898 ml, average blood loss was 1,108 ± 785 ml. All patients were admitted to the ICU post procedure. The average fluid balance during the OR was 9,481 ± 4,694 ml. Patients stayed in the ICU for an average of 6 ± 5.3 days. All patients survived the ICU stay. The duration of mechanical ventilation was 57 ± 83 hours, total fluid balance while in the ICU was 1,467 ± 3,399 ml. Hypomagnesemia was the most frequent electrolyte abnormalities in 79 (61%). Pleural effusions in 48 (37%), of which three patients only required drainage, Seven patients (5.6%) developed pneumonia, no patient required renal replacement therapy. Average hospital LOS was 33.7 ± 29 days. Only two patients died in the hospital. When the first 65 patients were compared with the last 64 patients, the duration of MV, ICU LOS and hospital LOS were all significantly shorter in the latter group (72 vs. 43 hours, 6.8 vs. 5.0 and 40 vs. 27 days respectively; *P *< 0.01 for all).

## Conclusion

With proper selection of patients, CRS with HIPEC can be done safely with no major complications. There is a significant reduction in ICU utilization and shorter hospital LOS with more experience in such procedure, suggesting a learning curve as well as better utilization of resources by referring such patients to a high-volume center.

